# Frequency-Dependent Altered Functional Connections of Default Mode Network in Alzheimer’s Disease

**DOI:** 10.3389/fnagi.2017.00259

**Published:** 2017-08-03

**Authors:** Youjun Li, Hongxiang Yao, Pan Lin, Liang Zheng, Chenxi Li, Bo Zhou, Pan Wang, Zengqiang Zhang, Luning Wang, Ningyu An, Jue Wang, Xi Zhang

**Affiliations:** ^1^The Key Laboratory of Biomedical Information Engineering of Ministry of Education, Institute of Biomedical Engineering, School of Life Science and Technology, Xi’an Jiaotong University Xi’an, China; ^2^National Engineering Research Center of Health Care and Medical Devices, Xi’an Jiaotong University Branch Xi’an, China; ^3^Department of Radiology, Chinese PLA General Hospital Beijing, China; ^4^Department of Neurology, Institute of Geriatrics and Gerontology, Chinese PLA General Hospital Beijing, China; ^5^Department of Neurology, Tianjin Huanhu Hospital Tianjin, China; ^6^Hainan Branch of Chinese PLA General Hospital Sanya, China

**Keywords:** frequency-dependent functional connectivity, functional network, default mode network, Alzheimer’s disease, resting state fMRI

## Abstract

Alzheimer’s disease (AD) is a neurodegenerative disorder associated with the progressive dysfunction of cognitive ability. Previous research has indicated that the default mode network (DMN) is closely related to cognition and is impaired in Alzheimer’s disease. Because recent studies have shown that different frequency bands represent specific physiological functions, DMN functional connectivity studies of the different frequency bands based on resting state fMRI (RS-fMRI) data may provide new insight into AD pathophysiology. In this study, we explored the functional connectivity based on well-defined DMN regions of interest (ROIs) from the five frequency bands: slow-5 (0.01–0.027 Hz), slow-4 (0.027–0.073 Hz), slow-3 (0.073–0.198 Hz), slow-2 (0.198–0.25 Hzs) and standard low-frequency oscillations (LFO) (0.01–0.08 Hz). We found that the altered functional connectivity patterns are mainly in the frequency band of slow-5 and slow-4 and that the decreased connections are long distance, but some relatively short connections are increased. In addition, the altered functional connections of the DMN in AD are frequency dependent and differ between the slow-5 and slow-4 bands. Mini-Mental State Examination scores were significantly correlated with the altered functional connectivity patterns in the slow-5 and slow-4 bands. These results indicate that frequency-dependent functional connectivity changes might provide potential biomarkers for AD pathophysiology.

## Introduction

Alzheimer’s disease is a progressive mental disorder associated with age and afflicts millions of elderly people with symptoms that evolve from initial memory loss to complete cognitive function decline. The disease changes not only the brain cortical structure but also its functional connectivity (Toussaint et al., 2014). Resting state functional magnetic resonance imaging (RS-fMRI) is widely used to detect functional abnormalities of brain networks that are impaired by progressive mental diseases, such as Alzheimer’s disease (Prodoehl et al., 2014; Griffanti et al., 2015; Iidaka, 2015; Khazaee et al., 2015; Son et al., 2015). Compared with non-resting fMRI techniques, RS-fMRI is easy to standardize and acquire and avoids performance-related variability of the fMRI task, and therefore, it may be more effective for identifying abnormalities associated with AD (Agosta et al., 2012).

In RS-fMRI, the default mode network (DMN) is the most-studied resting state network. It shows a high level of activity during rest and deactivates its performance during the cognitive tasks (Lin et al., 2011; Vidal-Pineiro et al., 2014; Raichle, 2015; Lin et al., 2016, 2017). Although the exact function of the DMN is not clear, it is involved in maintaining consciousness and monitoring internal and external stimuli (Gusnard and Raichle, 2001; Raichle et al., 2001; Greicius et al., 2003; Wicker et al., 2003). Previous studies have demonstrated that AD patients have significantly decreased functional connectivity of the DMN and that it is closely related with cognitive dysfunction (Li et al., 2002; Greicius et al., 2004; Rombouts et al., 2005; Wang et al., 2006, 2007; Allen et al., 2007; Supekar et al., 2008; Zhang et al., 2010; Zhou et al., 2010; Balthazar et al., 2014; Toussaint et al., 2014). Moreover, recent RS-fMRI studies showed enhance connectivity in a part of the DMN region in AD patients (Zhou et al., 2010).

In addition, previous studies have shown that fMRI signals are coupled with local field potentials (LFPs) (Logothetis et al., 2001; Lauritzen and Gold, 2003; Philippi et al., 2015). The different frequency oscillations of neuronal activity that produce LFPs are specifically linked with a variety of neural processes, such as plasticity, input selection, and consolidation (Buzsaki and Draguhn, 2004), and with cognitive functions, such as attention, memory and emotion (Knyazev, 2007), suggesting that the different RS-fMRI signals are correlated with different frequency ranges and even that each functional network corresponds to a specific frequency-specific rhythm (Mantini et al., 2007; Niu et al., 2014). In recent years, LFO with frequencies lower than 0.08 Hz have gained increased attention in fMRI studies (Demanuele et al., 2007). LFO contain coherent spontaneous low-frequency fluctuations during both resting and task-related conditions and reflect cortical cyclic modulations and long distance neuronal synchronization (Buzsaki and Draguhn, 2004; Vanhatalo et al., 2004; Balduzzi et al., 2008).

However, standard LFO, which only focus on the low frequency band (0.01–0.08 Hz) blood-oxygen-level dependent (BOLD) fluctuations, may have some limitations. Many studies have proposed that the BOLD signal in lower or higher frequencies is also physiologically significant (Wu et al., 2008; Baria et al., 2011; Niazy et al., 2011). The arbitrary selection of the frequency band may cause information loss in other frequencies. On the other hand, the band from 0.01 to 0.08 Hz covers a broad fluctuation range, and the information contained in potentially specific frequencies would be lost. Recently, Zuo and his colleagues extended the concept of RS-fMRI (Zuo et al., 2010). They divided RS-fMRI into four distinct frequency bands, slow-5 (0.01–0.027 Hz), slow-4 (0.027–0.073 Hz), slow-3 (0.073–0.198 Hzs), and slow-2 (0.198–0.25 Hz). Interestingly, they also found that the slow-5 frequency band tended to be present in cortical structures and that the slow-4 frequency band tended to be present in subcortical structures (Zuo et al., 2010). In addition, the slow-5 band exhibited higher power localized in the DMN than the slow-4 band (Zuo et al., 2010; Baria et al., 2011). Compared with standard LFO, this band allocation is more rational for depicting spontaneous brain activity at specific frequencies and also covers broader frequency bands. Recently, frequency-dependent changes in amplitude of low-frequency fluctuations (ALFF) and correlation patterns between functional connectivity and ALFF have been reported in MCI and in AD (Han et al., 2011, 2012; Liu et al., 2014; Mascali et al., 2015), even in other neurological disorders, such as Parkinson’s disease and schizophrenia (Zhang et al., 2013; Yu et al., 2014).

According to previous studies, we hypothesized that the functional connectivity of different DMN regions would be impaired in AD compared with normal cognitive (NC) healthy volunteers and that the functional connectivity pattern would be different in the different frequency bands. To test these hypotheses, we investigated the altered functional connectivity patterns in four different frequency bands and the standard LFO based on the DMN under no–task conditions in 35 patients with AD and 27 age-matched NC volunteers. In addition, we also explored the relationship between the *Z* score of the functional connectivity and Mini-Mental State Examination (MMSE) scores in different frequency bands.

## Materials and Methods

### Subjects

The subjects were recruited by the Chinese PLA General Hospital. We recruited the subjects from two sources: outpatients from the Chinese PLA General Hospital or recruitment through a website advertisement. Before the fMRI data acquisition, all participants underwent physical, psychological and laboratory examinations. The subjects were all right-handed and underwent a battery of neuropsychological tests: The MMSE, the Auditory Verbal Learning Test (AVLT), the Geriatric Depression Scale, the Clinical Dementia Rating (CDR) and the Activities of Daily Living (ADL) scale. Simultaneously, all subjects were evaluated by two senior neurologists and were not treated with any medication that might influence their cognition during the task.

The AD patient inclusion criteria were as follows: (1) AD was diagnosed using the ICD-10 criteria for AD, (2) CDR = 1 or 2, (3) not taking any nootropic drugs, such as anticholinesterase inhibitors, and (4) the ability to perform the neuropsychological tests and tolerate the MRI scanning. The AD patients also met the core clinical criteria for probable AD dementia. The NC criteria were as follows: (1) normal general physical status, (2) CDR = 0, and (3) no memory complaints.

We initially recruited 37 AD patients and 27 NC subjects. After excluding two subjects with large head motions (see Data Processing), 35 AD patients and 27 age- and gender- matched NC subjects were included for further analysis. Neuropsychological and Demographic details for the subjects are shown in **Table 1** and can be found in the previous studies (Wang et al., 2015).

**Table 1:** Demographics and neuropsychological characteristics.

	Control	AD	*P*
N	27	35	
Age(mean ± SD)	69.4 ± 6.7	71.8 ± 9.8	0.264
Sex(M/F)	15/13	12/23	0.124
MMSE score			
(Mean ± SD)	28.9 ± 1.0	19.3 ± 4.7	<0.001
CDR	–	1.3 ± 0.5	–
FD(mean ± SD)	0.27 ± 0.24	0.35 ± 0.30	0.220
DVARS(mean ± SD)	26.2 ± 9.1	30.52 ± 12.00	0.112


*According to two-sample *t*-test, no significant differences (*p* < 0.05) were observed in age or gender between the two groups. Significant differences were noted in MMSE scores between the two groups (*p* < 0.001). AD, patients with Alzheimer’s disease; NC, normal controls; MMSE, Mini Mental State Examination; CDR, Clinical Dementia Rate; FD, framewise displacement.*

### Image Acquisition

The MRI scans were performed at the Chinese PLA General Hospital, Beijing, China, with a 3.0 T GE MR system using a standard head coil. The subjects were instructed to do not think anything, to keep their eyes closed and to refrain from falling asleep during the scanning. A standard eight channel head coil was used to collect the data; ear plugs were used to reduce noise, and a comfortable foam pad was used to keep the head stable. T1-weighted images were collected before the resting fMRI. T1-weighted images were collected using a 3D fast-spoiled gradient echo sequence with the following parameters: TR = 7 ms, TE = 2.8 ms, flip angle = 8°, inversion time TI = 450 ms, image matrix = 256 × 256, voxel volume = 0.93 mm × 0.93 mm × 1.2mm, slice thickness = 1.2 mm, number of slices = 166. Resting-state fMRI data were acquired using an echo planar imaging (EPI) sequence with repetition time = 2000 ms, echo time = 30 ms, flip angle = 90°, matrix = 64 × 64, field of view = 220 mm × 220 mm, slice thickness = 3 mm, in plane spatial resolution = 3 mm × 3 mm and slice gap = 1 mm. Each volume was composed of 30 axial slices.

### Data Processing

Resting fMRI data were processed using the Analysis of Functional Neuroimaging (AFNI) software package (Cox, 1996) and the FSL (FMRIB’s Software Library^1^). For each subject, The first 4 volumes of each time series were discarded for magnetization equilibrium, and the motion correction was performed using 3D image realignment with the AFNI program 3dvolreg function, which uses a weighted least squares rigid-body registration algorithm. For each subject, the data were spatially smoothed using a Gaussian kernel of Full width at half maximum (FWHM) 6 mm. Next, we took the following steps: (1) remove the linear trends; (2) temporally bands pass filter the data according the five different frequency bands: LFO (0.01–0.08 Hz), slow-5 (0.01–0.027 Hz), slow-4 (0.027–0.073 Hz), slow-3 (0.073–0.198 Hz) and slow-2 (0.198–0.25 Hz). (3) remove several sources of spurious variance through linear regression by using FSL that include six rigid body motion correction parameters, the white matter signal and the cerebral spinal fluid (CSF) signal. Recently, the use of global signal regression is still under debate as a pre-processing step in resting-state fMRI analysis, and its use is not universally recommended (Murphy et al., 2009; Scholvinck et al., 2010). Further evidence suggests that the global component of fMRI fluctuations measured during the resting state is tightly coupled with underlying neural activity (Scholvinck et al., 2010). According the above reason, we do not remove the global whole brain signal. For further functional connectivity analysis, the each participant’s preprocessed fMRI data was then registered to the MNI152 standard template by using FSL’s linear registration algorithm (FLIRT). In addition, as participant motion can influence resting state functional connectivity, we further examined motion across different groups by using FSL. Framewise displacement (FD) and temporal derivative of the fMRI time series (DVARS) values were used to identify volumes in the fMRI time data. Previous study suggests that each FD exceed the 0.5 mm should be censored (Power et al., 2012). Two AD patients’ data were excluded from the dataset. To further compare head motion between AD and NC groups, we calculated the FD and DVARS between the two groups. Two sample *t*-test showed that FD and DVARS did not differ significantly between AD and NC groups (FD: *p* = 0.220; DVARS: *p* = 0.112; see **Table 1**).

**FIGURE 1:**
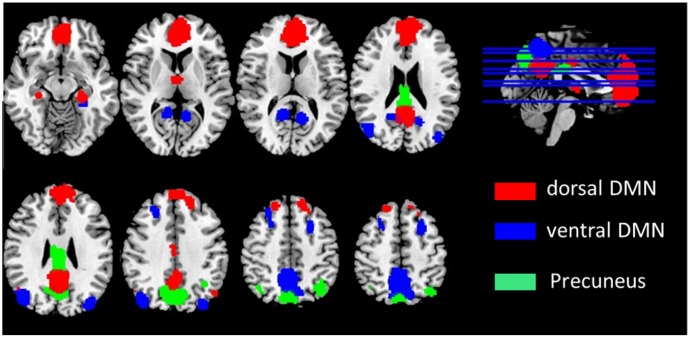
Defined ROIs in the default mode network.

**FIGURE 2:**
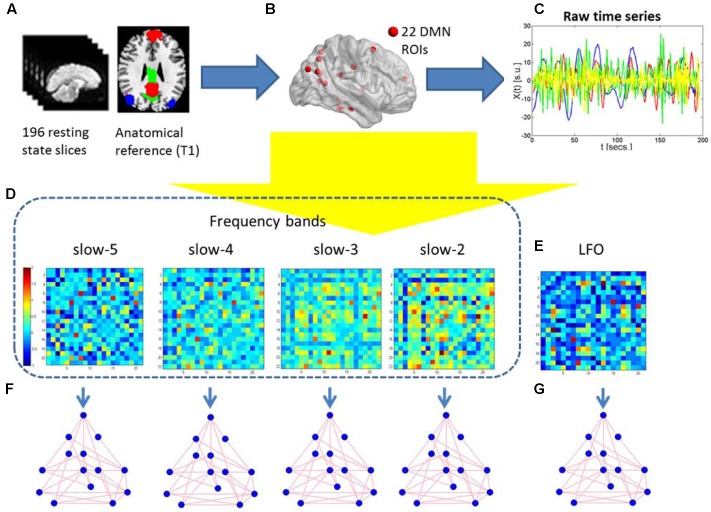
Schematic representation of the construction of frequency dependent default mode networks. **(A)** The 196 resting-state fMRI slices are co-registered to the anatomical T1 3D reference volume, and each voxel is mapped to one of the 22 ROIs in the MNI atlas. **(B)** The 22 DMN ROIs are obtained. **(C)** For each ROI, the raw time series of different frequency is retrieved using the 196 fMRI slices. **(D,E)** Five correlation matrices are obtained from the previous time series of the four different frequency bands and the standard LFO. They are Z-transformed to normalize correlation values across individuals. Warm (cold) colors in these matrices represent large (small) correlation values between ROIs. **(F,G)** Graph-theoretical measures of edge weight are obtained for each pair of DMN regions, to be analyzed using NBS.

After pre-processing, We used the DMN region of interest (ROI) template investigated from Shirer et al. (2012) study to extract the DMN time courses for the specific frequency bands (0.01–0.08 Hz, 0.01–0.027 Hz, 0.027–0.073 Hz, 0.073–0.198 Hz, and 0.198–0.25 Hz). The ROIs were downloaded from the website of Stanford Functional Imaging in Neuropsychiatric Disorders Lab^2^ (Shirer et al., 2012). The total 22 ROIs of the DMN in the brain were composed of nodes from the “dorsal DMN,” the “ventral DMN,” and the “precuneus” subnetworks of the atlas in **Figure 1** and **Table 2**. The average of all voxels in this ROI was calculated as the mean time series of the region, and the Pearson’s correlation coefficient between each pair of ROIs was computed as the strength of the functional connectivity. Fisher’s r-to-z transformation of the correlation coefficients was applied to improve the normality. **Figure 2** shows the schematic representation of the construction of frequency dependent DMN.

**Table 2:** Regions of interest (ROIs) coordinates.

Number	ROI	Brain region	BA	MNI Coordinate		
				
				*X*	*y*	*z*
1	dDMN_1_ROI	Medial frontal gyrus	10	0	49	12
2	dDMN_2_ROI	Left angular gyrus	39	-48	-73	32
3	dDMN_3_ROI	Right media frontal gyrus	8	18	38	51
4	dDMN_4_ROI	Precuneus	31	0	-57	30
5	dDMN_5_ROI	Cingulate gyrus	24	0	-17	35
6	dDMN_6_ROI	Right angular gyrus	39	48	-66	29
7	dDMN_7_ROI	Thalamus	–	-6	-6	3
8	dDMN_8_ROI	Left parahippocampal gyrus	35	-24	-37	-9
9	dDMN_9_ROI	Right parahippocampal gyrus	35	24	-21	-23
10	pDMN_1_ROI	Posterior cingulate	23	0	-35	28
11	pDMN_2_ROI	Precuneus	7	0	-76	38
12	pDMN_3_ROI	Left inferior parietal lobule	40	-39	-64	46
13	pDMN_4_ROI	Right inferior parietal lobule	40	39	-64	46
14	vDMN_1_ROI	Left posterior cingulate	30	-12	-62	10
15	vDMN_2_ROI	Left middle frontal gyrus	6	-27	6	59
16	vDMN_3_ROI	Left parahippocampal gyrus	37	-30	-39	-20
17	vDMN_4_ROI	Left angular gyrus	19	-36	-88	28
18	vDMN_5_ROI	Right posterior cingulate	30	15	-56	13
19	vDMN_6_ROI	Precuneus	7	-6	-61	56
20	vDMN_7_ROI	Right middle frontal gyrus	6	24	26	47
21	vDMN_8_ROI	Right parahippocampal gyrus	37	27	-33	-23
22	vDMN_9_ROI	Right angular gyrus	19	42	-79	28


*BA, Brodmann area; MNI, Montreal Neurological Institute.*

### Statistical Analysis

The coefficients resulted in a 22 × 22 matrix of weighted undirected graphs for each participant within each analyzed frequency band. Non-parametric permutation was implemented for inferential statistics to investigate group differences in the edge weights using the network-based statistic (NBS) toolbox which was described in detail previously (Zalesky et al., 2010). This toolbox was applied to the non-binarized connectivity matrices. A two-sample *t*-test statistics were applied to test whether the mean value of correlation of each pair of regions between groups was equal. This was repeated independently for each of the (22 × 21)/2 = 441 pairs of regions. The t statistic exceeding an uncorrected threshold of 3.2 (*P* < 0.05) were searched as connected components for the between-group difference interconnected networks in graph theory. Then permutation testing was applied to calculate the familywise error (FWE)-corrected *p*-value. For each permutation, the participants between the NC and AD group were exchanged randomly. The NBS was applied to the randomized data, and the size of the recorded largest network. 10,000 permutations were generated to yield an empirical null distribution for the size of the largest network. Finally, a corrected p value for a network of size k found in the original data was calculated as the proportion of permutations for which the largest network was greater than or equal to k. Using the size of the largest network in the permuted data ensured weak control of the FWE rate (Nichols and Holmes, 2002; Maris and Oostenveld, 2007). Next, independent-sample *t*-tests were applied to investigate the coefficients of the connections with significant differences using Statistical Product and Service Solutions (SPSS). To determine whether variation in the functional connectivity among the different frequencies was related to disease progression, a correlation analysis of the clinical MMSE and the functional connectivity was performed. The statistical significance level of *P* was less than 0.05.

## Results

### Altered Functional Connections between AD and NC Groups

**FIGURE 3:**
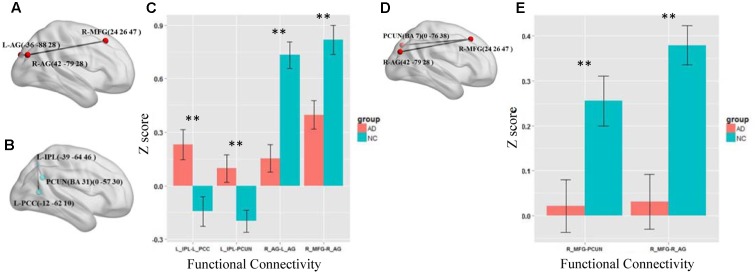
The altered functional connections in the slow-5 and slow-4 bands. **(A)** The decreased functional connectivity pattern of the AD group compared with the NC group in the slow-5 band. The functional connections of L_AG-R_MFG (L, means left side, R, means right side) and R_AG - L_AG are significantly decreased. **(B)** The increased functional connectivity pattern of the AD group compared with the NC group in the slow-5 band. The functional connections of L_IPL-PCUN, the L_IPL-L_PCC are significantly increased. **(C)** The differences in mean *Z* scores of the altered functional connections in the slow-5 band. **(D)** The decreased functional connectivity pattern of the AD group compared with the NC group in the slow-4 band. The functional connections of R_AG -R_MGF and PCUN-R_MFG are significantly decreased. **(E)** The differences in mean *Z* scores of the altered functional connections in the slow-4 band.

We first test the altered functional connections in the four specific frequency bands of slow-5, slow-4, slow-3, and slow-2. Only in the slow-5 and slow-4 bands, some functional connections in AD group are significantly changed compared with NC group by NBS. In the slow-3 and slow-2 bands, the NBS shows no significant difference between the AD and NC groups in the DMN. **Figures 3A–C** shows the differences in the brain region connections between the AD and NC groups in the slow-5 band. In the DMN, the NBS shows two significantly decreased functional connections in the AD group compared to the NC group (**Figure 3A**); One is between the right middle frontal gyrus (MFG) and the right angular gyrus (AG), and the other is between the right AG and the left AG (*P* < 0.01). However, the NBS also shows that two functional connections increased in the AD group (**Figure 3B**), one between the left inferior parietal lobule (IPL) and the precuneus (PCUN) and the other between the left IPL and the left posterior cingulate cortex (PCC) (*P* < 0.05). The composite *Z* scores of these connections also show significant differences (**Figure 3C**). **Figures 3D,E** illustrates the differences in the brain region connections in the slow-4 bands between the AD and NC groups. We also found two functional connections that significantly decreased in the AD group compared to the NC group in the DMN (**Figure 3D**), one is between the right MFG and the right AG, and the other one is between the right MFG and the PCUN (*P* = 0.01). The composite *Z* scores of these connections also show significant differences (**Figure 3E**). In the slow-4 band, none of the increased connection is found in the AD group compared to the NC group.

**FIGURE 4:**
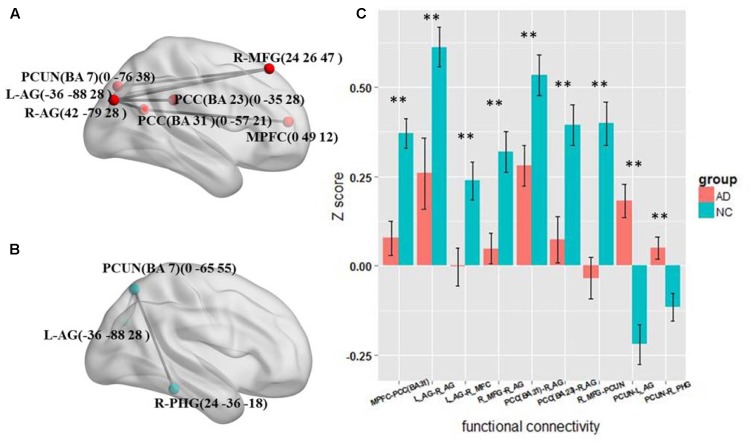
The altered functional connectivity in standard LFO band. **(A)** The decreased functional connectivity pattern of the AD group compared with the NC group in standard LFO band. The functional connections of MPFC -PCC(BA 31), L_AG - R_AG, L_AG-R_MFG, R_MFG - R_AG, PCC(BA 31)-R_AG, PCC(BA 23) -R_AG and R_MFG - PCUN are significantly decreased. **(B)** The increased functional connectivity pattern of the AD group compared with the NC group in standard LFO band. The functional connections of PCUN -L_AG and PCUN- R_PHG are significantly increased. **(C)** The differences in mean *Z* scores of the altered functional connections in the standard LFO band.

The results of the four frequency bands show that the altered functional connections are only in the slow-5 and slow-4 bands. The frequency range is similar to the standard LFO. Therefore we further tested the different connections between the AD and NC group in standard LFO. The result which is different from the slow-5 and slow-4 bands is shown in **Figure 4**. In standard LFO, there are seven decreased functional connections in AD group compared with NC group (**Figure 4A**), respectively between the MPFC and the PCC(BA 31), the right AG and the right AG, the left AG and the right MFG, the right MFG and the right AG, the PCC(BA 31) and the right AG, the PCC(BA 23) and the right AG, the right MFG and the PCUN (*P* < 0.01), and two increased functional connections (**Figure 4B**), respectively between the PCUN and the right AG, the PCUN and the right PHG (Para hippocampal Gyrus) (*P* < 0.01). The composite *Z* scores of these connections also show significant differences (**Figure 4C**).

### Correlations between Altered Connectivity and MMSE Scores

**FIGURE 5:**
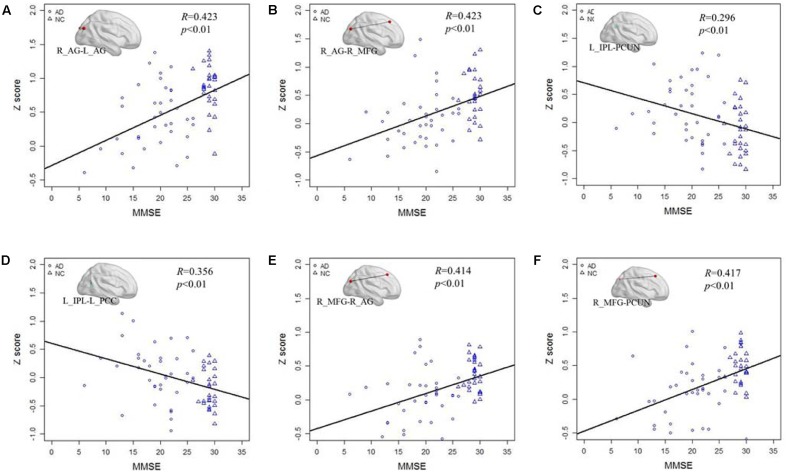
The correlation between the *Z* scores of the altered functional connections and the MMSE in the slow-4 and slow-5 bands. **(A,B)** The *Z* scores of the decreased functional connections, including L_AG - R_AG and R_AG -R_MGF, in AD group compared with NC group are positively correlated with the MMSE in the slow-5 band. **(C,D)** The *Z* scores of the increased functional connections, including L-IPL – PCUN and L_IPL-L_PCC, in AD group compared with NC group are negatively correlated with the MMSE in the slow-5 band. **(E,F)** The *Z* scores of the decreased functional connections, including R_AG-R_MFG and PCUN- R_MFG, in AD group compared with NC group are positively correlated with the MMSE in the slow-4 band.

After testing the frequency-dependent fingerprint of DMN in AD, we further explored the relationship between the *Z* score of the functional connectivity and MMSE scores in different frequency bands. **Figure 5** illustrates the correlation of the *Z* scores for altered functional connectivity and MMSE in slow-5 and slow-4 bands including the AD and NC groups. **Figures 5A,B** shows that, in slow-5 band, the *Z* scores of the connections between the right AG and the left AG (*R* = 0.423, *P* < 0.01) and between the right AG and the right MFG (*R* = 0.423, *P* < 0.01) which are decreased in AD group compared with NC are significantly positively correlated with the MMSE score. **Figures 5C,D** illustrates in slow-5 band, the decreased functional connectivity; the *Z* scores of the connections between the left IPL and the left PCC (*R* = 0.296, *P* < 0.01) and between the left IPL and the PCUN (*R* = 0.356, *P* < 0.01) are significantly negatively correlated with the MMSE score. The **Figures 5E,F** illustrates the correlation in the slow-4 band, the *Z* scores of the connections of the right MFG with the right AG (*R* = 0.414, *P* < 0.01) and of the right MFG with the PCUN (*R* = 0.417, *P* < 0.01) are also significantly positively correlated with the MMSE of the AD and NC group.

**FIGURE 6:**
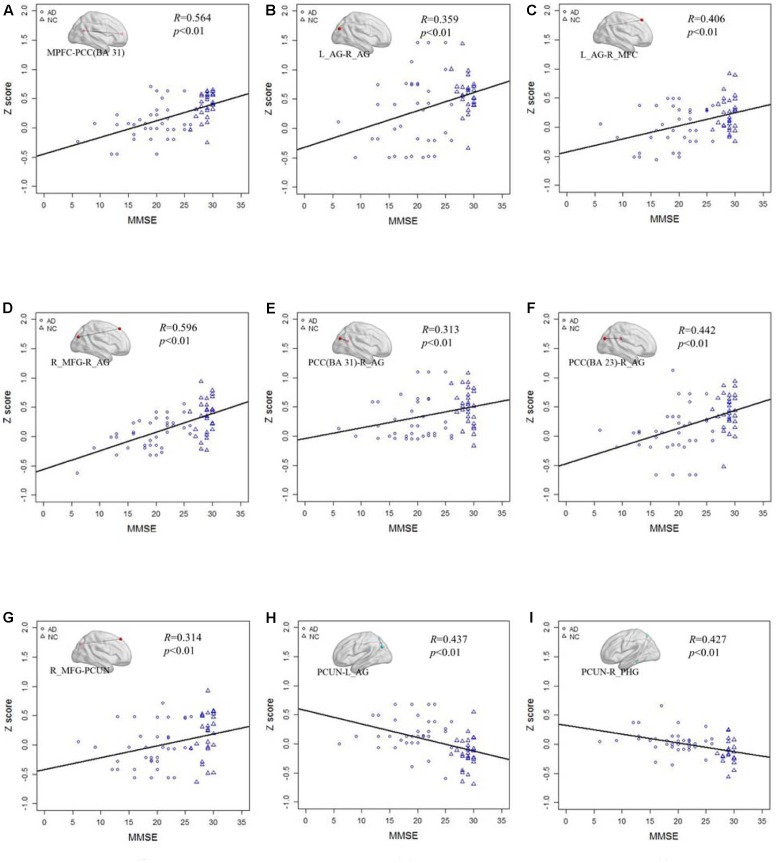
The correlation between the Z scores of the altered functional connections and the MMSE in the standard LFO band. **(A–G)** The *Z* scores of the decreased functional connections, including MPFC- PCC(BA 31), L_AG -R_AG, L_AG - R_MFG, R_MFG- R_AG, PCC(BA 31) - R_AG, PCC(BA 23) - R_AG and R_MFG -PCUN in AD group compared with NC group are positively correlated with the MMSE in standard LFO. **(H,I)** The *Z* scores of the increased functional connections, including PCUN - L_AG and PCUN - R_PHG, in AD group compared with NC group are negatively correlated with the MMSE in standard LFO band.

**Figure 6** shows the correlation of the *Z* scores and MMSE in standard LFO. **Figures 6A–G** illustrates the *Z* scores of the decreased functional connections between the AD and NC group in standard LFO were also significantly correlation with the MMSE score. **Figures 6H,I** show that the *Z* scores of the PCUN and the right AG (*R* = 0.437, *P* < 0.01), the PCUN and the right PHG (*R* = 0.427, *P* < 0.01) are also negatively correlated with the MMSE score.

## Discussion

This paper applied the NBS to study the frequency dependent characteristics of the functional connectivity among different brain regions in the DMN from the four different frequency bands and the standard LFO band between AD patients and NC healthy volunteers. We found in slow-4, slow-5 and standard LFO bands, several functional connections in DMN were significantly altered. We further investigated the *Z* scores of the significantly altered functional connections in different frequency bands were correlated with the MMSE scores.

### Frequency-Dependent Functional Connectivity Alterations of the Default Mode Network

The DMN is a key network in cognitive processes, especially in episodic memory, which is significantly affected in AD (Smith et al., 2009). In this study, our results in the standard LFO band by NBS showed several functional connections between different brain regions in DMN were decreased, while only two functional connections were increased. It was broadly consistent with the previous studies within the DMN in AD using multiple imaging techniques, and indicated AD might reduce the cognitive ability by impaired the functional connections in DMN (Brier et al., 2012; Dubois et al., 2014; Zhong et al., 2014; Ming et al., 2015). The separation results in the four frequency bands demonstrated the altered functional connections were only in the slow-4 and slow-5 bands. The NBS did not exhibit any altered functional connections in the slow-3 and slow-2 bands. It might be caused by the respiratory and cardiac fluctuation signals which influence the data analysis in the frequency range of 0.073–0.25 Hz (Cordes et al., 2001). Respiratory and cardiac fluctuation signals might create a common-mode signal in all extracted time series from all ROIs of the DMN. If the common signal was large enough, it would be significantly increased the correlation of the different brain regions and decrease the differences between the AD and NC groups in the slow-3 and slow-2 bands. The recent studies mainly focused in the slow-4 and slow-5 bands might also due to this reason (Han et al., 2011; Liu et al., 2014; Mascali et al., 2015). Furthermore, our results showed the decreased functional connections in standard LFO band were much more than in the slow-5 and slow-4 bands. The similar phenomenon was observed in the frequency- dependent patterns of coupling between correlation and amplitude of low-frequency fMRI fluctuations (Mascali et al., 2015). Indeed, the use of poorer information in slow-4 and slow-5 bands (nearly half of the spectrum) compared with the standard LFO band as well as the higher amount of noise which was present at lower frequencies, might result in a reduced statistical power, and caused the altered functional connections reduced (Mascali et al., 2015).

The results of altered functional connections in the slow-5 and slow-4 bands demonstrated that the functional connections of DMN were sensitive to the frequency range, and specific patterns of altered functional connections could be identified in the slow-5 and slow-4 bands. For instance, we showed the functional connection between the right MFG and the PCUN was decreased only in slow-4 band. Our results of decreased functional connections were consistent with the prior studies which pattern of intrinsic brain activity was sensitive to specific frequency bands (Di Martino et al., 2009; Hoptman et al., 2010; Zuo et al., 2010; Baria et al., 2011). Previous works suggested the synaptic delay and axonal conduction, along with physical constraints of the neural network main leaded to different oscillations, and the different oscillations might account for the frequency dependent functional connection in the two frequency bands (Buzsaki and Draguhn, 2004; Mascali et al., 2015). The functional connection between the right MFG and the right angular AG was a main altered functional connection because it was decreased in both slow-4 and slow-5 bands. The decreased connection between MFG and AG was observed in several previous researches (Wang et al., 2007, 2015; Zhong et al., 2014). The AG is proved in memory and visual perception and stores long-term visual memory (Miyashita, 1993), and the MFG is closely related with memory storage and retrieval (Cabeza et al., 2004), so the decreased functional connection between the right MFG and the right AG in both slow-4 and slow-5 band has an important effects on memory decline in AD patients. In addition, in the slow-5 band, we found two increased functional connections in AD group compared with NC group, the one was between the left IPL and PCC, and the other was between the left IPL and PCUN. Han et al. (2011) suggested the slow-5 band could be more sensitive in detecting abnormalities of spontaneous brain activity in the PCC/PCu and PHG in aMCI patients compared to the other bands, which provided further support for our results (Han et al., 2011). The increased functional connection in slow-5 bands reflect a compensation mechanism in AD patients, as reported in the previous studies that showed that increased connectivity was coupled with decreased functional connectivity (Wang et al., 2006; Supekar et al., 2008; Zhou et al., 2010).

In this study, it was interesting that, in both the slow-5 and slow-4 bands, the decreased functional connections were all long distance compared with the enhanced connectivity. This result was consistent with previous studies that have reported that the physical distances and the connectivity decay rates in the different frequency bands are positively correlated with the different brain regions (Wu et al., 2008; Wang et al., 2015). This phenomenon of the distance-frequency relationship might be attributed to the fact that different brain regions separated by longer distances communicate with each other through more synaptic steps caused a larger attenuation of correlation.

### The Correlation of Altered Functional Connectivity and Clinical Severity

Mini-mental state examination scores can reflect the global brain function in multiple cognitive domains and can be used to briefly scan AD patients and assess cognitive changes during the progression of AD (Wang et al., 2015). To assess whether the frequency-dependent alteration might hold promise as a biomarker for AD, we tested correlations between the *Z* scores and the MMSE clinical severity. The decreased functional connectivity in the slow-5 and slow-4 bands was significantly positively correlated with the MMSE scores, and the increased functional connectivity in these two frequency bands also was negatively correlated with the MMSE scores. Consistent with previous studies, the correlation between the MMSEs and the functional connectivity in different frequency bands indicates that the altered functional connectivity in different frequency bands might be a feature of the cognitive dysfunction caused by AD. Previous resting state fMRI studies showed that the DMN connectivity in the bilateral angular gyrus, the ventromedial prefrontal cortex and the right intraparietal sulcus are correlated with clinical index (Zhou et al., 2010). The significant correlation between the MMSE and the functional connectivity might indicate that the frequency-dependent functional connectivity is implicated in the decay of cognitive ability in AD. Additionally, the frequency-dependent functional connectivity differences between the AD and NC groups could be a potential marker for distinguishing AD patients from healthy subjects.

### Limitation and Future Directions

As is widely known, the brain network is a complex, interrelated and diverse system. It contains many neuronal networks, such as the executive control network, the salience network, and the dorsal attention network (Damoiseaux et al., 2006; Smith et al., 2009). Thus, a whole-brain network frequency-dependent analysis with different functional networks is needed in the future. In addition, previous studies have reported that the amyloid burden (Lim et al., 2014; Elman et al., 2016) or APOE e4 carriers (Trachtenberg et al., 2012) are closely related with disconnections of brain networks. Hence, further studies that combine fMRI with genotype or bio-analyses are necessary to identify the main reason behind the altered brain function. Finally, recent research has indicated the fMRI and EEG can be simultaneously detected, and the frequency-dependent analysis can be used in both datasets. We plan to analyze the combination of resting fMRI and resting EEG data in different frequency bands to further study the brain functional connectivity that is damaged by AD.

## Conclusion

In summary, the present study gives a frequency-dependent functional fingerprint of AD and the relationship between altered functional connection and disease severity. The results demonstrated that the altered functional connections were in the slow-4 and slow-5 bands, and the altered functional connections in different frequency bands were different. In addition, the decreased functional connections were long distance, and the increased connections were relatively short. Finally, the MMSE and the *Z*-scores of the altered functional connections were significantly correlated, indicating that the frequency-dependent altered functional patterns may be correlation with the progress of AD.

## Ethics Statement

This study was carried out in accordance with the recommendations of the approved guidelines Medical Ethics Committee of the Chinese PLA General Hospital with written informed consent from all subjects. All subjects gave written informed consent in accordance with the Declaration of Helsinki. The protocol was approved by the Medical Ethics Committee of the Chinese PLA General Hospital.

## Author Contributions

YL wrote the manuscript. LZ and CL analyzed the data. PL analyzed the data and revised the manuscript. HY, BZ, PW, and ZZ, collected the data. LW, NA, and JW revised the manuscript. XZ instructed the whole experiment.

## Conflict of Interest Statement

The authors declare that the research was conducted in the absence of any commercial or financial relationships that could be construed as a potential conflict of interest.

## Footnotes

1 www.fmrib.ox.ac.uk/fsl/fslwiki

2 http://findlab.stanford.edu/research
